# PatchChestCT: A patch-level spatial annotation dataset for nine abnormalities in chest CT

**DOI:** 10.1038/s41597-026-07793-0

**Published:** 2026-08-12

**Authors:** Yingtai Li, Hongchun Zhang, Mengwen Xu, Lidan Zhang, Najuan Lei, Yushuang Zhang, Xinli Zhu, Yan Lu, Wei Wei, Shaohua Kevin Zhou

**Affiliations:** 1 School of Biomedical Engineering, Division of Life Sciences and Medicine, University of Science and Technology of China (USTC), Hefei, Anhui 230026 China; 2 Center for Medical Imaging, Robotics, Analytic Computing & Learning (MIRACLE), Suzhou Institute for Advance Research, USTC, Suzhou, Jiangsu 215123 China; 3 Department of Radiology, The First Affiliated Hospital of USTC, Division of Life Sciences and Medicine, University of Science and Technology of China, Hefei, Anhui 230001 China; 4 Jiangsu Provincial Key Laboratory of Multimodal Digital Twin Technology, Suzhou, Jiangsu 215123 China; 5 State Key Laboratory of Precision and Intelligent Chemistry, USTC, Hefei, Anhui 230026 China; 6 Biomedical Basic Research Center (BBRC) of Jiangsu Province, Suzhou, Jiangsu 215123 China

## Abstract

The development of generalist artificial intelligence (AI) models for radiology is hindered by a lack of large-scale, three-dimensional (3D) imaging datasets with precise spatial annotations. While numerous datasets provide image-level labels for chest computed tomography (CT), these are insufficient for training models that can accurately localize findings. To address this gap, we present PatchChestCT, a large-scale, publicly available dataset for multi-abnormality localization in non-contrast chest CT. Using CT-RATE as the source cohort, PatchChestCT provides 3D patch-level annotations for nine clinically significant abnormalities across 2,201 physician-reviewed CT studies, with one reconstructed volume labeled per study. We introduce a token-aligned annotation scheme that is efficient, scalable, and naturally integrates with modern deep learning architectures like Transformers. To validate the dataset’s utility, we show that models trained with our patch-level labels achieved higher localization performance than weakly supervised baselines trained on image-level labels alone, across multiple architectures. PatchChestCT provides a public resource for training localization-aware models in chest CT.

## Background & Summary

Computed tomography (CT) is widely used in diagnostic imaging, offering high-resolution, three-dimensional insights into the human body. Chest CT, in particular, is indispensable in clinical practice, serving as a primary tool for the detection, diagnosis, and monitoring of a vast spectrum of thoracic conditions. It is routinely employed to identify critical findings across cardiovascular, pulmonary, and mediastinal systems. These abnormalities range from indicators of chronic disease, such as arterial and coronary wall calcification, to acute conditions like lung opacity and consolidation which can signify infections like pneumonia. Other important findings visible on chest CT include pericardial effusion (fluid around the heart), hiatal hernia, lymphadenopathy (enlarged lymph nodes, a potential sign of infection or malignancy), atelectasis (collapsed lung tissue), and bronchiectasis. The ability to visualize these diverse pathologies in detail makes chest CT a widely used modality.

However, this clinical utility has led to an increase in the volume of imaging data generated daily. A single chest CT examination can comprise hundreds or even thousands of individual axial slices, each requiring careful scrutiny by a radiologist. This growing imaging volume increases the interpretation burden, which can lead to fatigue, increase the risk of perceptual errors, and create bottlenecks in patient care. Consequently, there is a need for intelligent tools that can assist radiologists by streamlining their workflow and augmenting their diagnostic capabilities.

The recent and rapid advancements in artificial intelligence (AI), particularly the development of large-scale foundation models^1^, offer potential to address these challenges. The goal is to build generalist radiology AI systems^2^ that can interpret complex medical images with a high degree of competence, moving beyond narrow, single-task algorithms. However, the development of such sophisticated systems depends on access to large-scale, richly annotated datasets. While datasets for 2D imaging modalities like chest radiography are relatively well-established^3–7^, public resources for 3D volumetric imaging such as CT have been far more limited^8^.

To support reliable clinical use^9,10^, these generalist models must move beyond simple image-level classification (i.e., stating whether an abnormality is present in a scan) to deliver precise spatial localization-pinpointing exactly where the finding is located. Such localization capabilities are essential for several reasons. They enhance model transparency and explainability, allowing clinicians to verify the AI’s reasoning. They help mitigate the risk of models learning from spurious correlations or “shortcuts” in the data, a common pitfall of image-level classification learning^11^. Furthermore, accurate localization is important for downstream applications, from enabling automated measurements and quantitative analysis to facilitating interactive decision support systems^12^, improving radiology education^13^, and powering emerging applications that integrate large language models (LLMs) for the automated generation of structured radiology reports^14–16^.

Despite this clear need, creating volumetric localization datasets for CT is challenging. Manually delineating abnormalities slice-by-slice across a 3D volume is time-consuming and requires extensive effort from clinical experts, which explains the scarcity of such resources. To overcome this bottleneck, we introduce PatchChestCT, a large-scale, publicly available, 3D patch-level annotation dataset for multi-abnormality localization in non-contrast chest CT. The annotation scheme adopts a token-aligned granularity—a deliberate design choice that aligns with the input structures of modern AI architectures (e.g., CNN feature maps and ViT patch embeddings), thereby supporting the dataset’s practical utility for the research community and facilitating integration with multimodal LLMs^17^. In this paradigm, each volumetric scan is partitioned into a uniform grid of 3D patches, and annotations are applied at the patch level for nine clinically significant abnormality categories. This approach strikes a pragmatic balance between annotation efficiency and spatial precision, making it feasible to label large volumes of data while retaining clinically meaningful localization information. By providing this resource, PatchChestCT is intended to accelerate the development of localization-aware radiology AI models.

To situate our contribution, we compare PatchChestCT with contemporaneous resources that pursue related goals (Table 1). PatchChestCT targets non-contrast chest CT and provides openly available, token-aligned 3D patch localization (12 × 12 × 24 grid), complementing resources with different scopes. OmniAbnorm-CT^18^ is a whole-body abnormality dataset built from curated 2D key slices on Radiopaedia^19^, which limits voxel/volume-level tasks and requires paid licensing for source images, constraining accessibility. ReXGroundingCT^20^ offers sentence-level text-vision grounding labels, but segmentation is challenged for diffuse categories and at most three instances per finding per case result in partially annotated training data. In contrast, we exhaustively label all candidate patches across nine clinically important categories, extending beyond lung/pleura to cardiovascular and esophageal/gastroesophageal findings; to our knowledge, public localization data for arterial wall calcifications and hiatal hernia are scarce, and our annotations help fill this gap. This coarse but scalable strategy yields localization that is useful for screening/triage and is compatible with modern architectures (e.g., CNN feature maps, ViT patch embeddings), facilitating integration with multimodal LLMs. Looking ahead, combining our patch labels with foundation segmentation models such as Segment Anything^21,22^ or BiomedParse^23^ could enable a semi-automated pipeline to refine coarse localization into high-fidelity masks at scale.

**Table 1:** Positioning and comparison of chest CT annotation datasets.

Dimension	PatchChestCT	ReXGroundingCT	OmniAbnorm-CT
Core positioning / contribution	Token-aligned 3D patch localization	Sentence-level text-vision grounding	Whole-body abnormality dataset
Source data	CT-RATE (public)	CT-RATE (public)	Radiopaedia
Anatomical scope	Chest (incl. cardiovascular / mediastinal; 9 clinically important categories)	Chest (lung and pleura)	Multiple organ systems
Imaging planes	3D volumetric	3D volumetric	Selected 2D Slices
Data accessibility & cost	Annotations open; source images separately available under CT-RATE DUA	Annotations open; source images separately available under CT-RATE DUA	Annotations open; source images require paid licensing
Limitations	Coarse granularity; Single-institution cohort	Partially annotated; Single-institution data	Non-volumetric; Image access cost

## Methods

This section details the methodology used to create the PatchChestCT dataset. The process is organized into four main topics: (1) the source dataset and cohort selection, (2) the definition of target abnormalities and the annotation protocol, (3) the technical implementation of our patch-level annotation scheme, and (4) the annotation workflow and quality control procedures.

### Dataset source and cohort

We sourced non-contrast chest CT studies from the openly licensed CT-RATE^24^ dataset, a large-scale repository of non-contrast chest CT studies. The dataset is publicly available on the Hugging Face platform (https://huggingface.co/datasets/ibrahimhamamci/CT-RATE) and can be freely downloaded by any researcher after agreeing to the Data Usage Agreement posted on that page. The original CT-RATE dataset contains 25,692 chest CT studies from 21,304 individuals, with 20,000 used for training and 1,304 for testing, and these cases correspond to 50,188 reconstructed volumes and their associated narrative radiology reports, acquired between May 2015 and January 2023 at Istanbul Medipol University Mega Hospital in Turkey.

For our annotation efforts, we selected a subset of chest CT volumes from this collection. All selected volumes underwent a standardized preprocessing pipeline to ensure consistency. First, images were resampled to an in-plane resolution of 0.75 mm × 0.75 mm with a through-plane spacing of 1.5 mm. Subsequently, each volume was center-cropped to a uniform size of 384 × 384 × 192 voxels, a dimension chosen to consistently encompass the lungs and key surrounding mediastinal structures. Baseline demographic characteristics and imaging parameters for the selected cohort are summarized in Tables 2 and 3.

**Table 2:** Baseline Demographic Characteristics (subject level, by split).

Variable	Train (n = 2016)	Valid (n = 185)
Volumes with annotations, No.	2016	185
*Sex*		
Male, No. (%)	1138 (56.4)	109 (58.9)
Female, No. (%)	877 (43.5)	76 (41.1)
Unknown, No. (%)	1 (0.0)	0 (0.0)
*Age (y)*		
Mean ± SD	61.7 ± 14.4	60.3 ± 14.5
Median (IQR)	63 (52–72)	62 (49–71)
Range	18–102	22–89

Note.—Data are presented as numbers with percentages in parentheses or as means ± SDs/medians with IQRs, as indicated. Percentages are within split. One subject in the Train split lacked age (age available: Train n = 2015, Valid n = 185). IQR = interquartile range.

**Table 3:** CT Imaging Parameters and Scanner Information (subject level, by split).

Variable	Train (n = 2016)	Valid (n = 185)
*Pixel spacing (mm)*		
Mean ± SD	0.52 ± 0.17	0.54 ± 0.19
Median (IQR)	0.43 (0.37–0.69)	0.45 (0.37–0.74)
Range	0.27–1.01	0.28–0.87
*Z spacing (mm)*		
Mean ± SD	1.39 ± 0.20	1.36 ± 0.20
Median (IQR)	1.50 (1.25–1.50)	1.50 (1.25–1.50)
Range	0.75–2.00	0.75–1.50
*CTDIvol (mGy)*		
Mean ± SD	9.0 ± 5.0	8.3 ± 5.2
Median (IQR)	7.8 (4.8–11.1)	7.0 (4.6–10.4)
Range	0.8–34.6	2.0–33.4
*Scanner manufacturers (unique), No*.	4	4
*Manufacturer distribution, No. (%)*		
Philips	1266 (62.8)	113 (61.1)
Siemens Healthineers	576 (28.6)	66 (35.7)
SIEMENS	—	4 (2.2)
PNMS	92 (4.6)	—
Others	82 (4.1)	2 (1.1)

Note.—Data are numbers with percentages in parentheses unless otherwise indicated. CTDIvol = volume CT dose index. Manufacturer names are shown as recorded in DICOM (SIEMENS and Siemens Healthineers are listed separately, PNMS refers to Philips Neusoft Medical Systems).

### Ethics statement

This study analyzed fully de-identified, publicly available human chest CT data as described above. In accordance with institutional policies and applicable regulations, this did not constitute human subjects research and did not require institutional review board approval. No protected health information was accessed and no attempts at re-identification were made. Use of the data complied with all relevant terms of use and regulations.

### Target abnormalities and annotation protocol

We focused on annotating nine clinically significant abnormality categories that were defined in the original CT-RATE dataset: arterial wall calcification, pericardial effusion, coronary artery wall calcification, hiatal hernia, lymphadenopathy, atelectasis, lung opacity, consolidation, and bronchiectasis. Other important categories, such as pleural effusion and pulmonary nodules were excluded because labels for these findings can be obtained from widely used automated tools (e.g., TotalSegmentator^25,26^), so we prioritized under-served mediastinal/cardiac/gastrointestinal targets.

Our annotation protocol was designed for clarity and consistency. Annotators were instructed to visually inspect each CT volume and localize any present abnormalities corresponding to the nine target categories. The original labels in CT-RATE were derived automatically via natural language processing of radiology reports and thus may contain classification errors or lack spatial specificity. If a specific abnormality mentioned in the original report was not visually present in the scan, an empty label was recorded for that category, thereby correcting potential errors from the original text-based labels. To facilitate accurate identification of different pathologies, annotators were provided with an interface that allowed them to seamlessly switch between standard lung (W:1200, L:-400) and mediastinal (W:300, L:50) windowing presets.

### Patch-level annotation scheme

To enable efficient volumetric localization while minimizing the prohibitive costs of full voxel-wise segmentation, we developed and implemented a 3D patch-level annotation scheme^27^. This scheme is designed to balance annotation efficiency with the spatial precision required for training robust localization models.

Each preprocessed 384 × 384 × 192 voxel volume was conceptually divided into a 12 × 12 × 24 grid of non-overlapping cuboid patches. Each patch has a dimension of 32 × 32 × 8 voxels, corresponding to a physical volume of approximately 6.9 cm^3^. For each case, the annotation was performed on 24 evenly spaced reference axial slices (i.e., one every 8 slices through the z-axis). The labels assigned to a patch on a reference slice were then automatically propagated to its spatially adjacent patches in the volume, spanning ±4 neighboring slices along the z-axis.

This process results in a coarse 3D localization map where each patch is assigned a binary label for each of the nine abnormalities. A key advantage of this design is that each patch corresponds directly to a unit of representation in modern deep learning architectures, such as a feature vector in feature map of a Convolutional Neural Network (CNN) or the patch embeddings in a Vision Transformer. This “token-aligned” representation, a direct result of our efficient and scalable annotation workflow, also facilitates straightforward integration with multimodal large language models (MLLMs).

### Annotation workflow and quality control

The annotation process was carried out by a team of medical students who received standardized, comprehensive training on the annotation software and the specific guidelines for each abnormality category. To ensure a diverse selection of cases, each annotator was assigned a distinct range of study identifiers (e.g., cases 0001–5000) and was encouraged to preferentially include cases with visible abnormalities, while otherwise selecting cases randomly, within their assigned block.

A multi-stage quality control process was implemented to ensure the accuracy and reliability of the final labels. Every annotation produced was first visually reviewed by a single senior physician with extensive experience in chest CT interpretation. If any discrepancies or ambiguities were identified during this review, the case was flagged and re-annotated independently by two additional annotators. If the two additional annotators disagreed on the same case, the senior physician reviewed both annotations and made the authoritative final ruling. This multi-annotator review process served to resolve challenging cases and establish a consensus ground truth, ensuring a high standard for the entire dataset.

## Data Records

The PatchChestCT annotations generated in this study have been deposited in Zenodo as version 3 (v3)^28^ (10.5281/zenodo.19707049).

The Zenodo repository contains our newly created patch-level annotation files and the minimal CT-RATE volume identifiers required to link each annotation to its source volume. It does not include CT-RATE images, radiology reports, DICOM headers, subject-level clinical metadata, or original CT-RATE metadata tables. The annotations are released under a Creative Commons Attribution 4.0 International (CC BY 4.0) license, which applies only to the PatchChestCT annotation files and does not grant rights to any underlying CT-RATE data. Users must obtain the source CT volumes separately from the official CT-RATE repository^24^ and agree to its Data Usage Agreement.

**Fig. 1:**
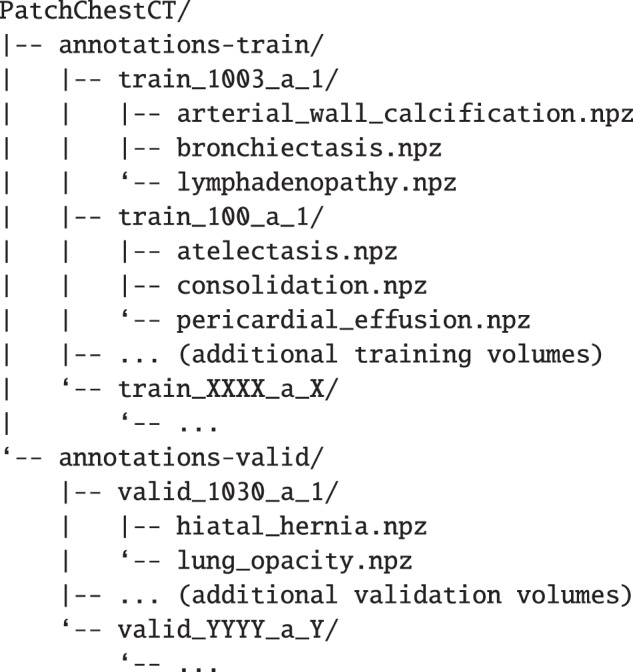
File structure of the PatchChestCT Zenodo deposit. Each subdirectory is named after the corresponding CT-RATE volume identifier. Only abnormalities present in a given volume have a corresponding .npz file; absent abnormalities are represented by the absence of a file.

The overall file organization is illustrated in Fig. 1. As shown, the dataset is divided into two primary directories: annotations-train/ for the training set and annotations-valid/ for the validation set. To maintain alignment with the official data splits of the source CT-RATE dataset, we omit a dedicated test set for our annotations. Each of these directories contains multiple subdirectories, where each subdirectory is named after a unique volume identifier from the original CT-RATE dataset (e.g., train_1003_a_1/).

Within each volume-specific subdirectory, one or more annotation files are stored in the compressed NumPy array format (.npz). This file organization is designed to be sparse: only abnormalities that are present in a given volume will have a corresponding.npz file. The filename of each.npz file explicitly names the abnormality class it represents (e.g., consolidation.npz, arterial_wall_calcification.npz). If no file for a particular abnormality exists within a volume’s folder, that volume is considered negative for that class.

Each individual.npz file contains a single binary data array, which can be accessed with the default key. This array is a tensor with a shape of (24, 12, 12), corresponding to the (Z, Y, X) dimensions of the patch grid for that volume. A value of 1 at a coordinate (z, y, x) indicates that the patch at that spatial location is positive for the abnormality specified by the filename. Conversely, a value of 0 indicates its absence in that patch.

## Technical Validation

To validate the quality, reliability, and utility of the PatchChestCT dataset, we conducted a multi-faceted evaluation. This validation encompasses four key areas: (1) the quality control protocol employed during annotation, (2) an inter-rater agreement study, (3) a statistical analysis of the dataset’s characteristics, and (4) expanded baseline modeling experiments to demonstrate the practical value of our patch-level labels for training localization models.

### Annotation quality control

The reliability of our dataset is anchored in a multi-stage quality control process. While the primary annotations were performed by trained medical students, annotations underwent a review by a senior physician with extensive experience in chest CT interpretation. This initial review served to identify and correct any obvious errors.

In cases where an annotation was deemed ambiguous or a discrepancy was noted, the volume was flagged for a secondary, more intensive review. These flagged cases were then independently re-annotated by two additional annotators. If the two additional annotators disagreed, the senior physician reviewed both annotations and made the authoritative final ruling. This multi-annotator consensus approach for challenging cases helped resolve ambiguities and ensure that the final labels achieve a high degree of clinical accuracy and consistency across the entire dataset.

### Inter-rater agreement

To provide quantitative evidence of annotation reliability, we conducted a dedicated inter-rater agreement study. We randomly sampled **100 volumes** from the annotated dataset. For each volume, two additional annotators independently performed the full patch-level annotation following the same protocol, without access to the existing labels or each other’s annotations.

Agreement was measured at two levels: (1) **Volume-level agreement**—whether a given abnormality category is present in the scan, quantified using Gwet’s AC1, a robust chance-corrected agreement coefficient that is less sensitive to prevalence imbalance than Cohen’s kappa; and (2) **Patch-level agreement**—spatial agreement on which patches are positive, quantified using the pairwise Dice similarity coefficient (DSC). The results are summarized in Table 4.

**Table 4:** Inter-rater agreement for the nine annotated abnormality categories.

Abnormality	Volume-level Gwet’s AC1	Patch-level DSC
Arterial wall calcification	0.882	0.607
Pericardial effusion	1.000	0.640
Coronary artery calcification	0.681	0.563
Hiatal hernia	0.958	0.214
Lymphadenopathy	0.891	0.413
Atelectasis	1.000	0.460
Lung opacity	0.922	0.382
Consolidation	1.000	0.643
Bronchiectasis	0.955	0.372
**Average**	**0.921**	**0.477**

Volume-level agreement was near-perfect for 8 of 9 categories (AC1 ≥ 0.88), with substantial agreement for coronary artery calcification (AC1 = 0.681); the mean AC1 across categories was 0.921, confirming reliable identification of abnormality presence/absence. Patch-level spatial agreement was highest for consolidation (DSC = 0.643), pericardial effusion (0.640), and arterial wall calcification (0.607), reflecting the anatomically well-defined appearance of these findings. Lower patch-level DSC for hiatal hernia (0.214) and bronchiectasis (0.372) reflects the spatially distributed and morphologically variable nature of these findings, where boundary patches are inherently ambiguous.

For hiatal hernia in particular, the low inter-rater patch-level DSC (0.214) coexists with a high volume-level AC1 (0.958): annotators agree well on whether the abnormality is present but may disagree on its exact patch boundaries. Results for this category in downstream benchmarking experiments should therefore be interpreted with caution.

We therefore recommend that PatchChestCT be used primarily as a **training resource**—to supply spatial supervision signals for learning patch-level localization—rather than as a strict benchmark for evaluating model performance.

To further assess the practical quality of the released annotations, the senior physician systematically reviewed all 185 validation-set volumes and applied corrections where necessary. The mean DSC between the original and expert-corrected annotations across all positively labeled patches was **0.886**, substantially higher than the blind inter-rater DSC of 0.477. This indicates that the released annotations are of practical quality, while the blind inter-rater DSC reflects the inherent spatial ambiguity of the annotation task itself.

### Dataset statistical analysis

**Fig. 2:**
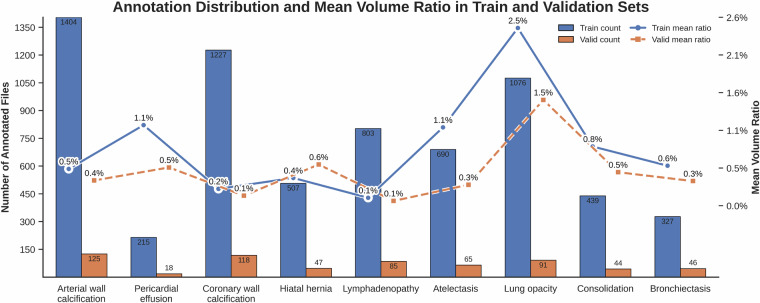
Dataset statistics. Bars show the number of annotated studies per category in the training and validation sets (blue/orange), and lines show the corresponding mean volume ratio (right axis; proportion of positive voxels/patches per scan). The plot highlights strong class imbalance and differences in spatial extent across categories; most classes have mean ratios below 1%, while lung opacity is notably higher (~2.5% in train).

A thorough analysis of the dataset’s statistical properties reveals important characteristics relevant for model training and evaluation. As summarized in Fig. 2, the distribution of abnormalities is highly imbalanced, which is representative of real-world clinical data. For instance, findings like arterial wall calcification and lung opacity are prevalent, whereas others are considerably less common.

Furthermore, the spatial extent of positive labels varies significantly across different abnormality categories. The mean volume ratio (the proportion of positive patches per scan) shows that some findings, such as lung opacity, tend to be diffuse and occupy a larger percentage of the volume. In contrast, most other findings are more focal, typically occupying less than 1% of the total patches in a volume. These statistics highlight the challenges–namely, class imbalance and variation in lesion size–that this dataset presents for developing robust AI models.

#### Subset representativeness

The annotated subset of 2,201 volumes (approximately 10% of CT-RATE) does not match the prevalence distribution of the full 50,188-volume cohort. Several categories are enriched (e.g., arterial wall calcification, coronary artery calcification) while others are underrepresented relative to the population baseline. Within their assigned blocks, annotators selected cases randomly while being encouraged to preferentially include cases with visible abnormalities, so as to ensure sufficient positive examples for training localization models. Prevalence matching to the source population is not required for this purpose; the relevant criterion for a localization training dataset is image feature diversity—covering the full range of lesion sizes, morphologies, and anatomical locations—not epidemiological prevalence. This diversity is inherited from CT-RATE’s 8-year collection span, multiple scanner platforms, and broad patient demographics (age range 18–102 y, balanced sex distribution; Tables 2 and 3).

### Utility for supervised model training

To empirically demonstrate the practical value of PatchChestCT, we conducted expanded baseline experiments comparing fully supervised models against weakly supervised counterparts across multiple architectures.

We evaluated three architectures: 3D ResNet-18 (R3D-18), Swin Transformer 3D (Swin3D-T)^29^, and Multiscale Vision Transformer (MViT-v2-S)^30^. For weakly supervised localization (WSL), we used two methods: (1) NoisyOR^31^, trained on image-level labels from the full 20,000-patient CT-RATE training set, and (2) Grad-CAM^32^, a widely used gradient-based saliency method applied to models trained with image-level supervision. For supervised localization, each architecture was trained directly on the 2,016 volumes with our patch-level annotations.

**Fig. 3:**
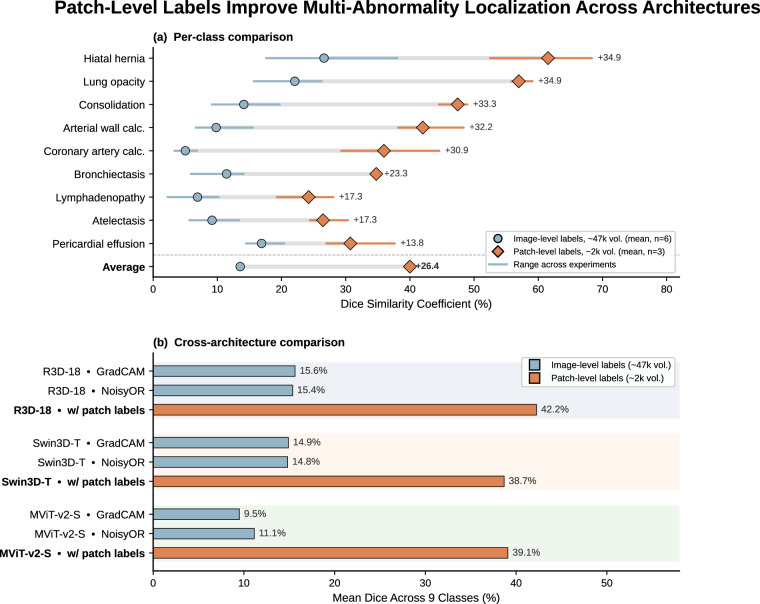
Multi-abnormality localization performance across architectures and supervision types. (**a**) Per-class comparison: dumbbell chart showing the mean Dice similarity coefficient (%) for image-level labels (blue circles, mean across 6 WSL experiments) versus patch-level labels (orange diamonds, mean across 3 architectures), with whiskers indicating the range. (**b**) Cross-architecture comparison: mean Dice (%) across all 9 classes for each architecture × method combination.

The results, shown in Fig. 3, showed higher localization performance with patch-level supervision. Across all three architectures and both WSL methods, patch-level supervision yielded substantially higher mean DSC (38.7–42.2%) than all WSL approaches (9.5–15.6%), a consistent gap of more than 23 percentage points. The best model trained on PatchChestCT labels achieved a mean Dice similarity coefficient (DSC) of 42.2%, a substantial increase over the best WSL result of 15.6% DSC (+26.6%). The performance gains were consistent across all nine abnormality categories, with particularly large improvements for arterial wall calcification (+32.2%), coronary artery wall calcification (+30.9%), and lung opacity (+34.9%). This result suggests that the performance gain derives from the patch-level annotation itself rather than any specific model choice. This experiment provides evidence that image-level supervision alone is insufficient for reliable spatial localization and indicates that the detailed, patch-level ground truth in PatchChestCT provides useful supervision for training localization-capable radiology AI systems.

## Usage Notes

The PatchChestCT dataset is primarily designed to support the development and standardized evaluation of models for multi-abnormality localization in 3D chest CT. The patch-based labels are particularly well-suited for training modern deep learning architectures, including 3D Convolutional Neural Networks and Vision Transformers. Beyond fully supervised training, this dataset can serve as a resource for research into semi-supervised or mixed-supervision learning, by combining our detailed patch-level ground truth with the much larger collection of image-level labels available in the full CT-RATE dataset.

An important consideration for practitioners is the trade-off between annotation granularity and dataset scale. Weakly supervised methods benefit from much larger pools of inexpensive image-level annotations (e.g., the full 20,000-patient CT-RATE training set). Although our results show that such weak supervision is insufficient for reliable spatial localization (mean DSC 9.5–15.6% vs. 38.7–42.2%), image-level labels remain advantageous for classification-oriented tasks. Our dataset is therefore designed to complement, not replace, image-level labels; mixed-supervision learning—combining both signals—is a promising direction.

Users should be aware of the dataset’s inherent limitations. The coarse patch granularity (32 × 32 × 8 voxels, ~6.9 cm^3^) is a deliberate trade-off enabling scalable annotation. For spatially diffuse abnormalities (lung opacity, consolidation, atelectasis) the patch-level labels capture affected regions at the patch level; for focal findings (arterial/coronary wall calcifications, lymphadenopathy) they provide localization to ~2.4 cm × 2.4 cm × 1.2 cm—clinically useful for directing attention but insufficient for precise boundary delineation. Users requiring sub-patch precision should consider our labels as spatial priors that can be refined using foundation segmentation models such as SAM^21,22^ or BiomedParse^23^. Another limitation is the sparse nature of annotation along the z-axis, where labels were generated from 24 evenly spaced reference slices. Each 3D patch spans only 8 voxels (12 mm) along the z-axis, and the reference slice is positioned at its center (±4 slices); for the nine target abnormalities—most of which are anatomically contiguous structures—a finding occupying any portion of a 12 mm axial extent is very likely to appear on the central reference slice.

Finally, the data originate from a single institution. While the CT-RATE cohort exhibits substantial internal diversity—spanning 8 years, multiple scanner manufacturers, and 20,000 patients—it inherently reflects the practice patterns of one clinical center. Because our annotations target fundamental structural anomalies (e.g., the visual presence of consolidation or calcification) rather than high-level aggregate diagnoses, the learned visual features are expected to generalize to other populations. Nonetheless, models trained on this dataset should undergo external, multi-institutional validation before clinical use.

## Data Availability

The PatchChestCT annotations are publicly archived on Zenodo as version 3 (v3)28 (10.5281/zenodo.19707049) under a CC BY 4.0 license. The archive contains the annotation files and minimal CT-RATE volume identifiers needed for linkage; no CT-RATE images or radiology reports are included. Source CT-RATE volumes must be obtained separately from CT-RATE under its Data Usage Agreement.

## Code availability

We provide the Python source code used for the baseline experiments in a public GitHub repository to ensure full reproducibility: https://github.com/SadVoxel/PatchChestCT. This repository contains all necessary scripts to work with the PatchChestCT dataset. The key components include:

• A custom PyTorch data loader designed to handle our sparse, directory-based .npz file structure.

• The complete implementation for technical validation of data utility for supervised model training.

The code is written in Python and primarily relies on the PyTorch and NumPy libraries. The repository’s README file provides detailed instructions. The GitHub repository provides code and documentation for using the annotations, including the CC BY 4.0 license for PatchChestCT annotation files and the requirement to obtain source CT-RATE data separately under its Data Usage Agreement.
